# l-DNA-Based Catalytic Hairpin Assembly Circuit

**DOI:** 10.3390/molecules25040947

**Published:** 2020-02-20

**Authors:** Adam M. Kabza, Jonathan T. Sczepanski

**Affiliations:** Department of Chemistry, Texas A&M University, College Station, TX, 77843, USA; akabza@tamu.edu

**Keywords:** catalytic hairpin assembly (CHA), strand-displacement reaction, peptide nucleic acid, l-DNA, microRNA

## Abstract

Isothermal, enzyme-free amplification methods based on DNA strand-displacement reactions show great promise for applications in biosensing and disease diagnostics but operating such systems within biological environments remains extremely challenging due to the susceptibility of DNA to nuclease degradation. Here, we report a catalytic hairpin assembly (CHA) circuit constructed from nuclease-resistant l-DNA that is capable of unimpeded signal amplification in the presence of 10% fetal bovine serum (FBS). The superior biostability of the l-DNA CHA circuit relative to its native d-DNA counterpart was clearly demonstrated through a direct comparison of the two systems (d versus l) under various conditions. Importantly, we show that the l-CHA circuit can be sequence-specifically interfaced with an endogenous d-nucleic acid biomarker via an achiral peptide nucleic acid (PNA) intermediary, enabling catalytic detection of the target in FBS. Overall, this work establishes a blueprint for the detection of low-abundance nucleic acids in harsh biological environments and provides further impetus for the construction of DNA nanotechnology using l-oligonucleotides.

## 1. Introduction

The straightforward programmability of Watson–Crick (WC) base pairing interactions makes nucleic acids an ideal material for engineering nanoscale structures and devices. Underlying the operation of most dynamic DNA nanotechnology is the toehold-mediated strand-displacement reaction [1,2,3]. During this process, a single-stranded overhang region (referred to as a “toehold”) on an otherwise complementary DNA duplex initiates recognition and invasion by a third DNA strand, which ultimately displaces the original strand not containing the toehold region. Owing to its simplicity, DNA strand-displacement reactions have been widely used for engineering molecular devices, including motors and walkers [4,5,6,7], reconfigurable DNA nanostructures [8,9], and logic circuits [10,11,12]. Importantly, such devices can be easily interfaced with regulatory nucleic acids (e.g., mRNAs and microRNAs) via WC base pairing [13,14], making them particularly well suited for applications in bioengineering and disease diagnosis.

Due to the low abundance of most nucleic acid biomarkers in biological fluids and tissues, analytical application of DNA nanodevices often requires signal amplification. Thus, it is not surprising that significant effort has gone into engineering non-enzymatic DNA amplifier circuits that can detect and amplify nucleic acid signals based on strand-displacement mechanisms [15]. Examples of DNA amplifiers include entropy driven catalytic circuits [16], hybridization chain reactions [17] and various DNAzyme-based systems [18]. Perhaps one of the most versatile DNA amplifiers is the catalytic hairpin assembly (CHA), originally developed by Pierce and coworkers [5]. CHA circuits utilize a pair of complementary DNA hairpins to achieve isothermal, enzyme-free, signal amplification. Spontaneous hybridization between the two hairpins is kinetically hindered because the complementary sequence domains are embedded within the hairpin stems. However, in the presence of a target input strand, one of the hairpins can be opened via toehold-mediated strand-displacement reactions, which in turn enable the assembly (hybridization) of both hairpins. During this assembly process, the input strand is displaced from the annealed hairpin complex, allowing it to initiate further rounds of hairpin opening and assembly. CHA circuits provide rapid and efficient signal amplification with minimal background and fast turnover rates. Consequently, CHA circuits have been adapted to a variety of analytical applications, including the detection of quantification of therapeutically relevant nucleic acids in vitro and in living cells [3,19,20].

Despite the promise of DNA amplifiers in low-abundance biomarker discovery and clinical diagnosis, the straightforward implementation of such devices in harsh biological environments remains challenging for several reasons. In particular, natural DNA is susceptible to nuclease-mediated degradation and non-specific interactions with other nucleic acids and proteins, both of which can lead to high background and/or poor signal amplification in living cells [13]. Although modifications of the 2′-OH group of the ribose sugar (e.g., 2′-O-methyl ribonucleotides [21,22] and locked nucleic acids [20,23]), as well as the phosphate backbone modifications (e.g., phosphorothioates) [24], can confer nuclease stability, such modified oligonucleotides still have the potential for off-target hybridization, and in some cases, cellular toxicity [25]. Importantly, the majority of modified oligonucleotides have altered kinetic and thermodynamic properties relative to native DNA, making it very difficult to apply established design principles to the development of amplifier circuits composed of such polymers. Therefore, developing robust DNA amplifiers capable of catalytic amplification in biological environments remains an important challenge.

**Figure 1:**
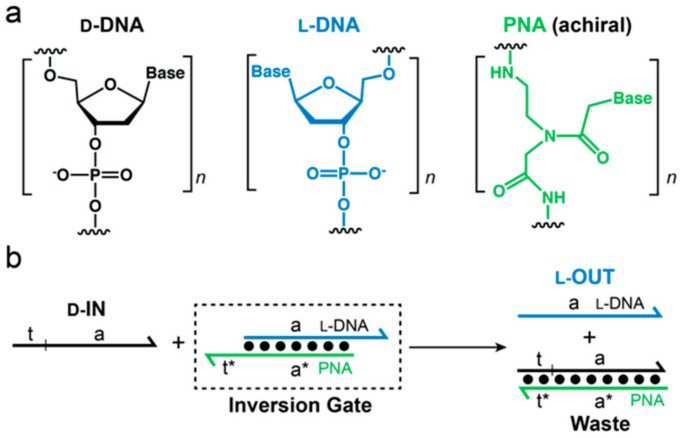
(**a**) Three types of nucleic acids used in this work. d-DNA (black), l-DNA (blue), and peptide nucleic acid (PNA) (green) are distinguished by color throughout the text; (**b**) Inversion Gate. The toehold domain (t*) resides on the achiral PNA strand in the l-DNA/PNA heteroduplex (Inversion Gate). Therefore, the d-input can still bind to the inversion gate (via t and t*) and displace l-OUT. In this way, the sequence information in domain (**a**) has become inverted.

Recently, we challenged the idea of classical nucleic acid modifications by employing l-DNA, the enantiomer of natural d-DNA, in DNA circuit design. l-DNA is an ideal oligonucleotide analog because it is completely nuclease resistant, yet has identical kinetic and thermodynamic properties as its native counterpart, d-DNA [26]. Furthermore, L-oligonucleotides are incapable of forming contiguous WC base pairs with the native polymer [27,28]. Thus, l-DNA avoids off-target interactions with myriad of cellular nucleic acids. Nevertheless, we previously reported a method to interface specific nucleic acid targets with l-DNA using strand-displacement reactions [29]. This approach, termed “heterochiral” stand-displacement, employs an achiral peptide nucleic acid (PNA) in order to transfer sequence information between oligonucleotide enantiomers (Figure 1). The reaction involves of a complex between an achiral PNA strand and an l-DNA strand (l-OUT). We refer to this complex as an “inversion gate”. Importantly, a single-stranded toehold domain t* resides on the achiral PNA strand, which facilitates binding of a d-input strand (d-IN) to the inversion gate via t/t* and subsequent displacement of the incumbent l-DNA strand (l-OUT) or vice versa. In this way, any D-oligonucleotide input, including disease biomarkers, can be sequence-specifically interfaced with bio-stable l-DNA nanodevices or circuits, providing a promising approach for overcoming several key limitations of using such devices in cells or other harsh biological environments. For example, we recently used this approach to interface oncogenic microRNAs with an l-RNA-based fluorescent biosensor, enabling real-time imaging of microRNA expression levels in living mammalian cells [30]. Despite the potential advantages of l-DNA/RNA-based devices, a heterochiral l-DNA amplifier circuit has not previously been reported.

Here, we report the design and implementation of the first l-DNA amplifier circuit capable of detecting native D-oligonucleotides. The amplifier consists of a single PNA/l-DNA inversion gate, the output of which initiates an l-DNA-based CHA circuit allowing for the detection of the native d-input, microRNA-155 (miR-155), at sub-stoichiometric concentrations. We show that both d-DNA and l-DNA versions of the optimized amplifier circuit behave similarly, achieving signal amplification under physiological conditions. However, only the l-DNA amplifier retains faithful operation in the presence of 10% FBS. Overall, this work demonstrates that CHA circuits constructed from l-DNA, together with a heterochiral inversion gate, provide a robust and straightforward approach for detection low-abundance nucleic acids within harsh biological environments.

## 2. Results and Discussion

**Figure 2:**
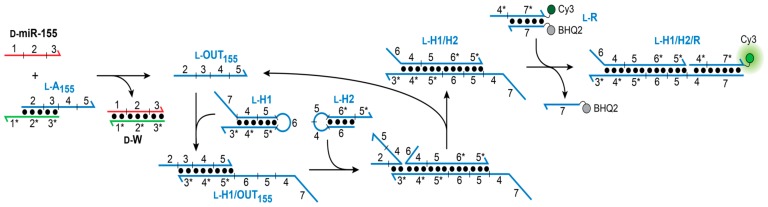
Schematic illustration of the heterochiral l-CHA circuit. Sequences of all strands are listed in Table S1. d-MiR-155 RNA is colored red.

Our goal was to design a CHA circuit comprised of l-DNA that could ultimately be interfaced with disease-relevant nucleic acid biomarkers. The target chosen for this study was miR-155, a prototypical oncogenic miR associated with various malignancies [31]. The overall heterochiral CHA amplifier circuit is illustrated in Figure 2. The reaction between d-miR-155 and the miR-155-specific inversion gate (l-A_155_) results in the displacement of l-OUT_155_, which subsequently initiates the opening of hairpin l-H1 via toehold domain 3*. The newly exposed single-stranded domains on l-H1 (5 and 6) then hybridize to hairpin l-H2 (via toehold-domain 5*), triggering the formation of product duplex l-H1/H2 and displacement of l-OUT_155_ from l-H1. The recycled l-OUT_155_ strand can then go on to initiate further rounds of hairpin l-H1 opening and catalysis. The reaction can be monitored by a reporter complex (l-R) that reacts with domain 4 on hairpin l-H1 (via 4*) only after opening of l-H1. The choice of target immediately restricts the overall circuit design because the sequence of the inversion gate (A_1_) must have partially complementarity with sequence with d-miR-155 (domains 1–3). In turn, the toehold domain (3*) on hairpin l-H1 is also dependent on the sequence of miR-155. However, beyond domain 3, the remaining sequences for both hairpins H1 and H2, as well as the fluorescent reporter duplex l-R may be chosen as required for the particular application of the system.

**Figure 3:**
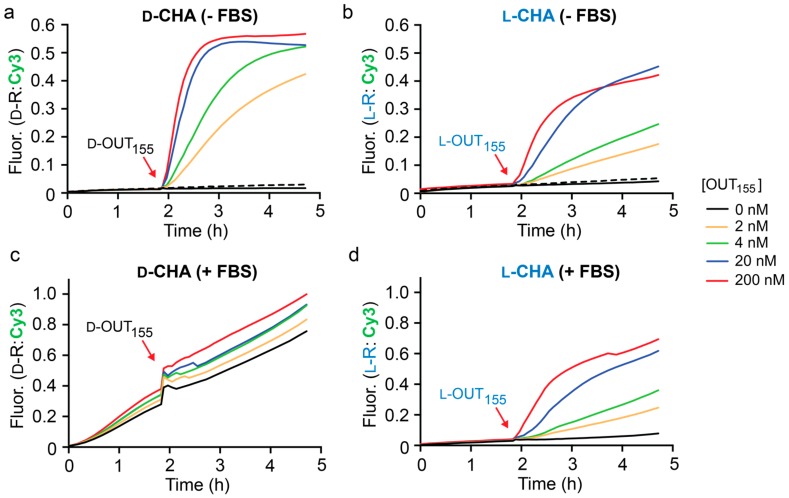
Fluorescence monitoring (Cy3) of CHA reactions in the absence (**a**,**b**) and presence (**c**,**d**) of 10% fetal bovine serum (FBS). All reaction mixtures contained 200 nM hairpins (H1 and H2) and 200 nM reporter complex (R) in the indicated stereochemistry, along with either 0% or 10% FBS, 50 mM KCl, 20 mM NaCl, 1 mM MgCl_2_, and 25 mM TRIS (pH 7.6). Reactions were initiated with the indicated concentration of either d- or l-OUT_155_ and were carried out at 37 °C. CHA reactions initiated with a scrambled input OUT_s_ (200 nM) are indicated by dotted lines. Fluorescence (Fluor.) in all figures is reported in units such that 0.0 and 1.0 are the fluorescence of the quenched and activated reporter complex, respectively, at 200 nM. Average fluorescence data from triplicate experiments is plotted.

Given that the sequence of the inversion gate (l-A_155_) was essentially fixed by miR-155, we initially focused our attention on identifying optimal sequences for CHA hairpins H1 and H2. Following principles originally established by Ellington and coworkers [32], we designed and tested a series of hairpins by varying the length and nucleotide composition of complementary domains (domains 4–6). To increase the efficiency of this process, all experiments were carried out using d-DNA and hairpin assembly reactions were monitored by native gel electrophoresis (Figures S1 and S2). Ultimately, we identified a pair of hairpins, d-H1 and d-H2 (Table S1), which retained high stability under simulated physiological conditions (i.e., 50 mM KCl, 20 mM NaCl, 1 mM MgCl_2_, pH 7.6, 37 °C), yet rapidly assembled into complex d-H1/H2 the presence of the initiator strand (d-OUT_155_). Therefore, all further studies were based on these two hairpins. As shown in Figure 3a, the rate of the CHA reaction between d-H1 and d-H2 was highly dependent on the concentration of initiator d-OUT_1_, as monitored by fluorescence (Cy3) using reporter d-R. When 2 nM d-OUT_155_ was added, i.e., 100-fold lower concentration that the hairpins and reporter, 40% maximal fluorescent signal was observed after 3 h, representing 20-fold signal amplification. This data indicates that this CHA circuit can provide rapid and efficient signal amplification under physiological conditions. We note that despite the presence of stoichiometric initiator (200 nM d-OUT_155_), the CHA circuit failed to achieve the maximum fluorescence signal for the reporter complex (d-R), indicating incomplete hairpin opening and/or reporter activation. Importantly, a negligible fluorescence signal was observed for up to 2 h prior to the addition of d-IN_1_ to the reaction (Figure 3a), confirming that hairpins d-H1 and d-H2 do not spontaneously hybridize in the absence of the initiator strand. Furthermore, a scrambled version of d-OUT_155_ (d-OUT_S_) failed to initiate the reaction, demonstrating the specificity of this CHA circuit (Figure 3a).

Having confirmed the proper operation of the CHA circuit using d-DNA components, we prepared l-DNA versions of the same components (l-OUT_155_, l-H1, l-H2, and l-R) using solid-phase phosphoramidites chemistry (Table S1). Overall, the l-DNA CHA circuit behaved similarly to its d-DNA counterpart (Figure 3b), but with a somewhat reduced rate of signal amplification. Initial rates for the d- and l-CHA reactions in the presence of stoichiometric initiator (200 nM) were calculated to be 54.02 ± 2.64 min^−1^ and 24.78 ± 1.0 min^−1^, respectively. We attribute this discrepancy to potential differences in oligonucleotide quality, as well as other experimental limitations, such as pipetting and concentration errors. Nevertheless, the l-CHA circuit generated ~20% maximal fluorescent signal in the presence of 2 nM l-OUT_155_, representing ~10-fold signal amplification. To the best of our knowledge, this represents the first example of a nucleic acid amplifier comprised entirely of mirror-image l-DNA.

With both d- and l-versions of the CHA circuit in hand, we compared their performance in the presence of 10% fetal bovine serum (FBS) as a model biological environment (Figure 3c,d). We have previously shown that both l-DNA and l-RNA are stable in 10% FBS for long periods of time [30,33]. As before, the circuit components were allowed to incubate for 2 h prior to the addition of the in initiator strand OUT_155_. As expected, the D-CHA circuit was rapidly degraded during the 2 h pre-incubation period, as evident by an initiator-independent gain in fluorescence signal (i.e., leak) (Figure 3c). Moreover, addition of the initiator strand (d-OUT_155_) to the D-CHA circuit after 2 h failed to promote any meaningful signal amplification relative to background (i.e., no initiator). In contrast, the presence of 10% FBS had little effect on the operation of the l-DNA version of the CHA circuit (Figure 3d). Negligible fluorescence signal was observed during the 2 h pre-incubation period, indicating that the l-DNA circuit components, and in particular hairpins l-H1 and l-H2, remained intact in the presence of 10% FBS. This was confirmed by gel electrophoresis (Figure S3). Importantly, initiation of the l-CHA reaction using l-OUT_155_ resulted in a concentration dependent fluorescence response, again reaching ~20% maximal signal in the presence of 100-fold lower concentration of l-OUT_155_ relative to reporter after 3 h. Overall, the fluorescent data obtained for the l-CHA circuit in the presence of 10% FBS (Figure 3d) closely mirrored data obtained in its absence (Figure 3b), demonstrating that complex biological matrixes do not significantly interfere with the operation of l-DNA-based CHA reactions.

**Figure 4:**
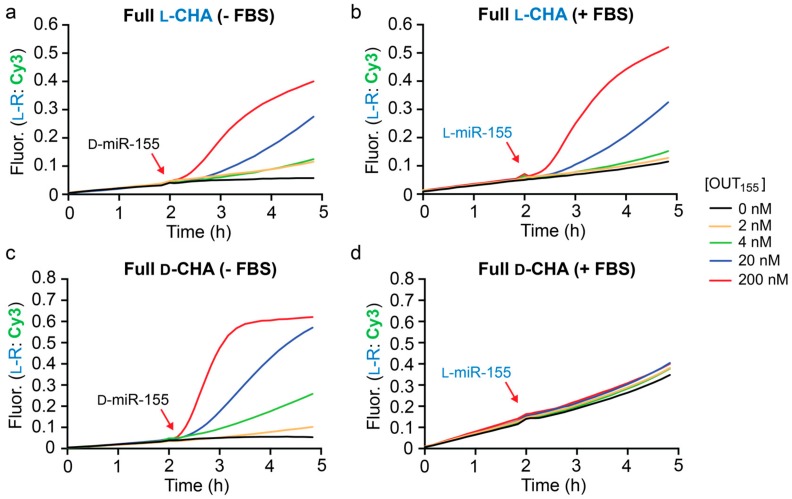
Fluorescence monitoring (Cy3) of the full heterochiral CHA circuit in the absence (**a**,**c**) and presence (**b**,**d**) of 10% FBS. Reaction conditions are identical to those described in Figure 3, except that 200 nM inversion gate A_155_ was also included. Reactions were initiated with the indicated concentration of either d- or l-miR-155 as indicated and were carried out at 37 °C. Average fluorescence data from triplicate experiments is plotted.

The l-CHA reactions depicted in Figure 3 were initiated directly using either d- or l-OUT_155_. However, our ultimate goal was to utilize an l-CHA circuit to detect D-miR-155, which required an inversion gate be placed upstream of the l-DNA hairpins (Figure 2). As discussed above, the sequence of the inversion gate (l-A_155_) was dictated by the sequence of D-miR-155 (Table S1), and was designed such that binding of D-miR-155 to the achiral PNA toehold domain (1*) resulted in displacement of the incumbent strand l-OUT_155_, which subsequently initiates the CHA reaction via domains 3/3*. We assembled and tested the full heterochiral CHA circuit depicted in Figure 2, which consisted of l-A_155_, l-H1, l-H2, and l-R_1_. All concentrations of D-miR-155 input tested resulted in the generation of a fluorescence signal that was greater than background (Figure 4a). However, it was clear that these reactions were significantly slower than the corresponding CHA reactions that were directly initiated with l-OUT_155_ (Figure 3b). This likely reflects the relatively slow kinetics of the heterochiral strand-displacement reaction between D-miR-155 and l-A_155_ [29]. Despite the reduced rate, however, the heterochiral amplifier was still capable of modest signal amplification (~3–5-fold).

**Figure 5:**
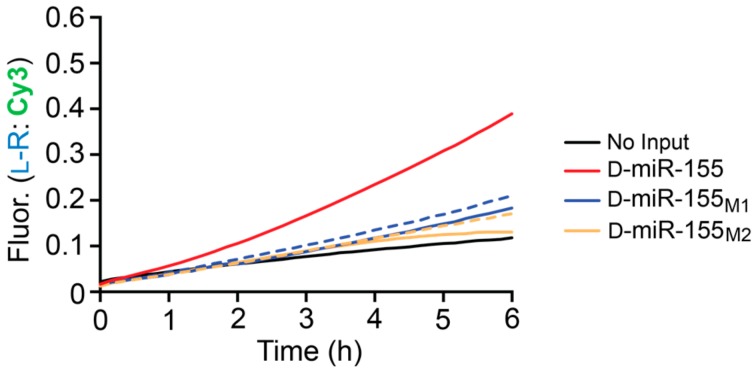
Mismatch discrimination by the full heterochiral CHA circuit. Reaction conditions are identical to those described in Figure 4. Reactions were initiated with either 20 nM input (solid lines) or 200 nM input (dotted lines) and were carried out at 37 °C.

To test for selectivity, we attempted to initiate the heterochiral CHA reaction with D-miR-155-derived inputs containing either one or two mismatches (D-miR-155_M1_ or D-miR-155_M2_, respectively) in the toehold-binding domain 1 (Figure 2 and Table S1). These reactions were carried out for an extended period of time (6 h) to ensure that any small amount of non-specific initiation by the mismatched substrates could be detected through CHA amplification. At 20 nM input concentrations (10-fold less than reporter), both mismatched substrates resulted in significantly less signal generation then D-miR-155 (Figure 5), which achieved ~4-fold amplification during the reaction. Increasing the concentration of both mismatched substrates by 10-fold did not greatly increase the signal generated by the system, allowing the CHA circuit to detect D-miR-155 (20 nM) in the presence of excess mismatched target RNA (200 nM). In all cases, the signal generated by the single and double mismatched substrates were similar. Overall, this data indicates that the heterochiral CHA circuit can discriminate against sequences containing a single mismatch, at least within the toehold domain.

Finally, we tested the full heterochiral CHA circuit in 10% FBS. The circuit maintained functionality in 10% FBS (Figure 4b), although with somewhat reduced sensitivity towards D-miR-155 due to a higher background fluorescence signal. This suggests possible circuit leakage due to an uninitiated reaction between the inversion gate (l-A_1_) and hairpin l-H1 in serum. An l-RNA version of miR-155 (l-miR-155) was employed as the input during these experiments to avoid nuclease degradation prior to circuit activation. The full l-CHA circuit remained intact during the 2 h pre-incubation period in the presence of 10% FBS and treatment with 20 nM l-miR-155 resulted in the generation of a fluorescence signal equivalent to ~3-fold amplification. Not surprisingly, incubation of the d-DNA version of the full CHA circuit (d-A_155_, d-H1, d-H2, and d-R_1_) in 10% FBS resulted in significant circuit leakage during the 2 h pre-incubation period and failed to activate upon the addition of l-miR-155 input (Figure 4c), further highlighting the advantage of l-DNA. While further optimization is needed, the above results demonstrate that the heterochiral l-CHA amplifier circuit described herein can be made compatible with the detection of low-abundance nucleic acids in complex biological samples.

## 3. Conclusions

In summary, we have successfully demonstrated a l-DNA CHA amplifier. The l-CHA circuit exhibited superior stability and catalysis in 10% FBS relative to it d-DNA counterpart, and when integrated with a heterochiral inversion gate, was capable of signal amplification in response to a d-RNA target (miR-155). To the best of our knowledge, this represents the first example of a nucleic acid amplifier comprised of mirror-image l-DNA. Given the resistance of l-oligonucleotides to cleavage by nucleases, we anticipate that this approach will further expand the utility of DNA amplifiers within harsh biological environments, enabling exciting analytical applications currently not achievable using systems based on native d-DNA. For example, having previously shown that heterochiral strand-displacement reactions can be used to interface disease-associated miRs with l-oligonucleotide-based biosensors in living cells [30], l-CHA circuits may provide a route towards ultrasensitive and selective miR detection for clinical early diagnosis. Towards this goal, it will be exciting to examine the operation of l-CHA amplifiers in living cells.

## 4. Materials and Methods

### 4.1. General

Oligonucleotides were either purchased from Integrated DNA Technologies (Coralville, IA, USA) or prepared by solid-phase synthesis on an Expedite 8909 DNA/RNA synthesizer (ThermoFisher Scientific, Waltham, MA, USA). Synthesizer reagents, D-nucleoside phosphoramidites, and Cy3 phosphoramidites were purchased from Glen Research (Sterling, VA, USA). l-nucleoside phosphoramidites were purchased from ChemGenes (Wilmington, MA, USA). Black Hole Quencher 2 resins were purchased from LGC Biosearch Technologies (Petaluma, CA, USA). Peptide nucleic acids (PNA) were purchased from PNA Bio Inc. (Newbury Park, CA, USA) at 99.9% purity and were not purified further. All other reagents were purchased from Sigma Aldrich (St. Louis, MO, USA).

### 4.2. Oligonucleotide Purification and Assembly

Unmodified D-oligonucleotides were purchased from IDT. All l-oligonucleotides were synthesized in house following the manufacturer’s recommended procedures, and completed l-oligonucleotides were deprotected using a 1:1 mixture of aqueous ammonium hydroxide and aqueous methylamine for 30 min at 65 °C. All oligonucleotides were purified by 20% denaturing polyacrylamide gel electrophoresis (PAGE, 19:1 acrylamide:bisacrylamide). Purified material was excised from the gel and eluted overnight at 23 °C in Buffer EB (200 mM NaCl, 10 mM EDTA, and 10 mM Tris pH 7.5). The solution was filtered to remove gel fragments, and the eluent was precipitated with ethanol. Duplex components (A_155_ and R) for each CHA circuit were assembled via a hybridization titration approach in order to achieve an ideal 1:1 ratio of the corresponding strands. Here, one strand was held constant at 1 μM while the concentration of the second strand was varied across a narrow range around 1 μM (0.80–1.20 µM in 0.05 µM increments). All hybridization mixtures contained the appropriate amount of each strand, 300 mM NaCl, 1 mM EDTA, 10 mM Tris (pH 7.6) and were heated to 90 °C for 3 min then cooled slowly to room temperature over 2 h. The extent of hybridization was quantified by 20% native PAGE (19:1 acrylamide:bisacrylamide) after staining with SYBR Gold (ThermoFisher Scientific, Waltham, MA, USA). Only those mixtures having an ideal 1:1 ratio of strands (i.e., no single-stranded oligonucleotide remained) were used further. The ideal 1:1 ratio of hairpins H1 and H2 strands were determined in a similar manner.

### 4.3. Fluorescence Monitoring of CHA Reactions

CHA reactions were monitored using a GloMax Discover multi-well plate reader from Promega Corp. (Madison, WI, USA). All reaction mixtures contained 200 nM each H1, H2, and reporter R in the indicated stereochemistry, along with either 0% or 10% FBS, 50 mM KCl, 20 mM NaCl, 1 mM MgCl_2_, and 25 mM TRIS (pH 7.6). For reaction containing the full CHA circuit (Figure 4), 200 nM inversion gate A_155_ was also included. Reactions were prepared to a final volume of 20 µL and transferred to a 384-well black-walled microplate. After the 2 h pre-incubation at 37 °C, the indicated concentration of initiator was added (OUT_155_ for CHA only reactions or miR-155 for the full circuit) and the reaction allowed to proceed for 3 h. Fluorescence was monitored with excitation/emission wavelengths at 520/580–640 nm (bandpass filter: Cy3). Data was normalized to a control representing the maximum achievable signal using Equation (1):

(1) $$F_{n}=\frac{F-F_{0}}{F_{c}-F_{0}}$$

where *F_n_* is the normalized fluorescence intensity, *F* is the measured fluorescence, *F_0_* is the fluorescence of the quenched reporter, and *F_c_* is the fluorescence of the activated reporter at each time a measurement was taken.

### 4.4. Monitoring of Heterochiral Strand-Displacement Reactions by Native PAGE

In some instances, CHA reactions were analyzed by 20% native PAGE (19:1 acrylamide:bisacrylamide) (Figure S2). Reactions were prepared as described above and incubated for 2 h at 37 °C before an aliquot was taken (5 μL) and loaded onto a running gel. Native gels were run at 140 volts for at least 6 h at 23 °C before being imaged as described above.

## Supplementary Materials

The following are available online at https://www.mdpi.com/1420-3049/25/4/947/s1. Figure S1. Schematic illustration of the d-DNA and l-DNA versions of the full CHA circuit; Figure S2. Native PAGE (20%; 19:1 acrylamide:bisacrylamide) analysis of the CHA reaction; Figure S3. Denaturing PAGE (20%; 19:1 acrylamide:bisacrylamide) analysis of hairpins H1 and H2 in the presence of different amounts of FBS; Table S1. Names, sequences, and chirality of all oligonucleotides used in this work.
