# Supplementary material for: l-DNA-Based Catalytic Hairpin Assembly Circuit
Source: Molecules. 2020 Feb 20;25(4):947. doi: 10.3390/molecules25040947 (PMC7070954; doi:10.3390/molecules25040947)
Supplement: Supplementary file 1 [file molecules-25-00947-s001.pdf]

Supplementary Information

L-DNA Catalytic Hairpin Assembly Circuit

Adam M. Kabza[1] *and Jonathan T. Sczepanski*[1,*]

[1]Department of Chemistry, Texas A&M University, College Station, Texas 77843

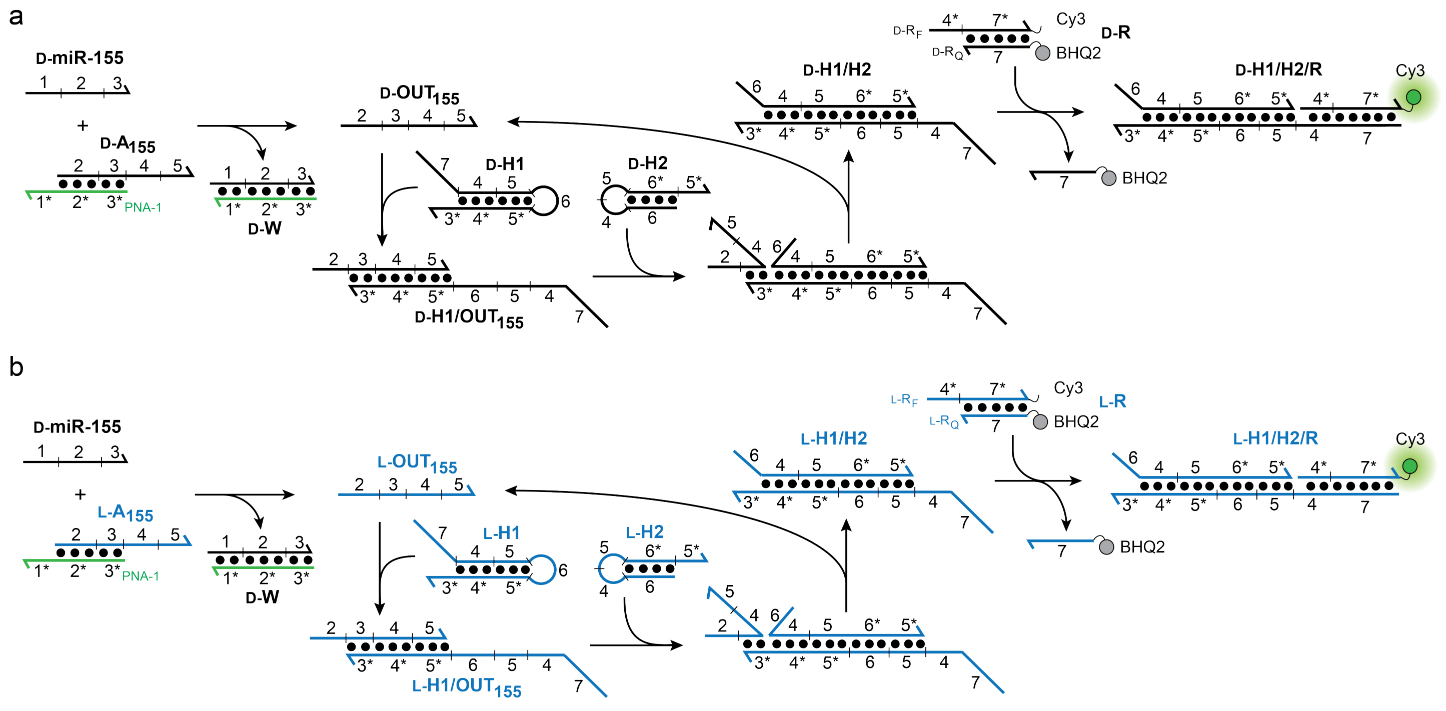

Figure S1. Schematic illustration of the D-DNA (a) and L-DNA (b) versions of the full CHA circuit. Sequences of all strands are listed in Table S1.

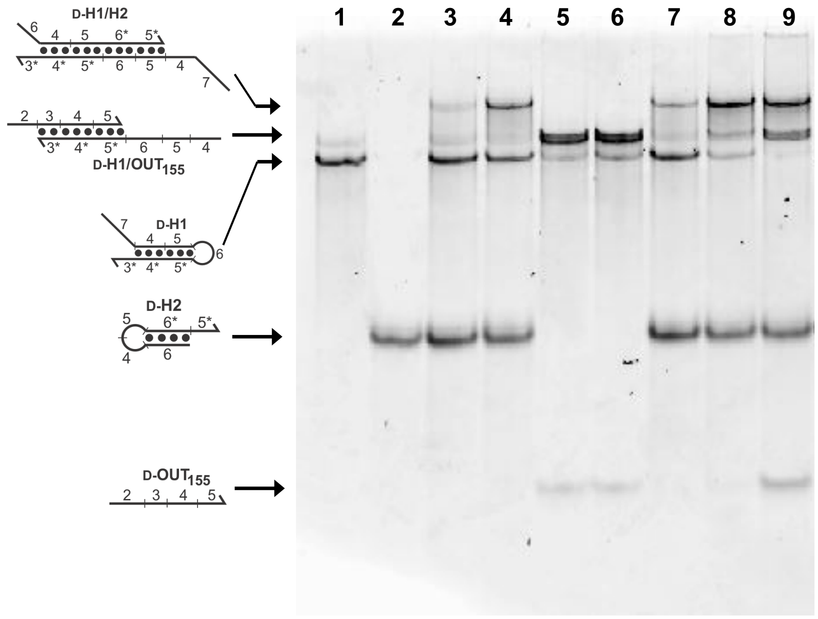

Figure S2. Native PAGE (20%; 19:1 acrylamide:bisacrylamide) analysis of the CHA reaction. Reaction conditions are identical to those described in Figure 3 in the main text. Lane 1: D-H1; lane 2: D-H2, lane 3: D-H1 and D-H2; lane 4: D-H1 and D-H2 after annealing; lane 5: D-H1 and D-OUT_{155}; lane 6: D-H1 and D-OUT_{155} after annealing; lane 7: D-H1 D-H2, and D-OUT_{155} (4 nM); lane 8: D-H1 D-H2, and D-OUT_{155} (40 nM); lane 9: D-H1 D-H2, and D-OUT_{155} (200 nM). Unless stated otherwise, the concentration of all components was 200 nM.

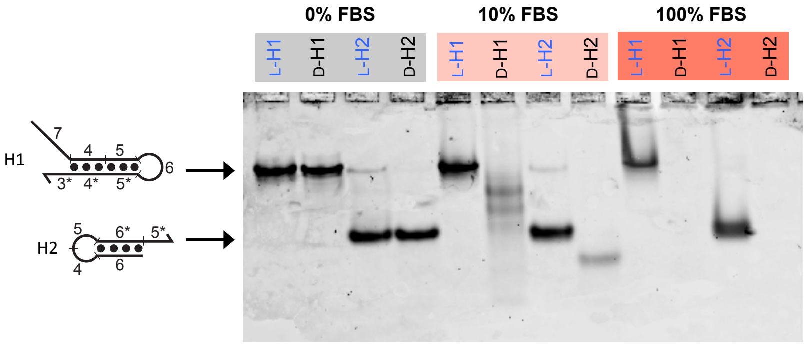

Figure S3. Denaturing PAGE (20%; 19:1 acrylamide:bisacrylamide) analysis of hairpins H1 and H2 in the presence of different amounts of FBS. The indicated hairpin (200 nM) was incubated with either 0%, 10%, or 100% FBS for 6 hours at 37 °C in a reaction mixture containing 50 mM KCl, 20 mM NaCl, 1 mM $MgCl_2$, and 25 mM TRIS (pH 7.6).

SI Table 1. Names, sequences, and chirality of all oligonucleotides used in this work. D-DNA (black), D-RNA (red), L-DNA (blue), and PNA (green) are indicated by color. 3Cy3 = cyanine 3 dye; 5BHQ2 = Black Hole Quencher 2. Refer to Figure S1.

Sequence Name	Sequence Identity 5' → 3'	Oligonucleotide Stereochemistry
D-H1	ACGTTACCTGCCCTATAGGATCGAACTGGTAAGATGTGTACTACCAGTTC GATCCTATCACGAT	D
L-H1	ACGTTACCTGCCCTATAGGATCGAACTGGTAAGATGTGTACTACCAGTTC GATCCTATCACGAT	L
D-H2	AGATGTGTACATAGGATCGAACTGGTAGTACACATCTTACCAG	D
L-H2	AGATGTGTACATAGGATCGAACTGGTAGTACACATCTTACCAG	L
D-R_F	ATCCTATAGGGCAGGTAACGTC/3Cy3/	D
L-R_F	ATCCTATAGGGCAGGTAACGTC/3Cy3/	L
D-R_Q	/5BHQ2/GACGTTACCTGCCCTAT	D
L-R_Q	/5BHQ2/GACGTTACCTGCCCTAT	L
D-OUT_{155}	GCTAATCGTGATAGGATCGAACTGGTA	D
L-OUT_{155}	GCTAATCGTGATAGGATCGAACTGGTA	L
D-OUT_S	GCTATAGCACATAGGATCGAACTGG	D
L-OUT_S	GCTATAGCACATAGGATCGAACTGG	L
PNA-1	CCTATCACGATTAGCATTAA	Achiral
D-miR-155	UUAAUGCUAAUCGUGAUAGGGUU	D
D-miR-155_{M1}	UUAAU**C**CUAAUCGUGAUAGGGUU	D
D-miR-155_{M2}	UU**T**AU**C**CUAAUCGUGAUAGGGUU	D
L-miR-155	UUAAUGCUAAUCGUGAUAGGGUU	L
